# DNA Methylation Studies in Saliva of Patients with Sjögren’s Syndrome

**DOI:** 10.31138/mjr.32.2.176

**Published:** 2021-06-30

**Authors:** Panagiota Karagianni, Efstathia K. Kapsogeorgou, Athanasios G. Tzioufas, Andreas V. Goules

**Affiliations:** Department of Pathophysiology, School of Medicine, National and Kapodistrian University of Athens, Greece

**Keywords:** Methylation, epigenetics, DNA, saliva, Sjögren’s syndrome

## Abstract

Sjögren’s syndrome (SS) is a relatively common systemic autoimmune disease of unknown aetiology, although genetic, hormonal, immunologic, and environmental factors are thought to be involved in disease pathogenesis. It is also termed “autoimmune epithelitis”, and afflicts mainly the epithelial structures of salivary and lachrymal glands, through periepithelial lymphocytic infiltration responsible for the occurrence of dryness symptoms. Sjögren’s syndrome (SS) is also characterised by B cell hyperactivity as reflected by the presence of hypergammaglobulinemia and the production of autoantibodies, which seems to be associated with the presence of ectopic germinal centres within the inflamed minor salivary glands. Chronic antigenic stimulation may lead to expansion of B cell autoreactive clones with rheumatoid factor activity, and additional molecular events mediate malignant transformation into non-Hodgkin’s lymphomas of B cell origin. Therefore, the interaction between the immune cells of the inflammatory infiltrate and the salivary epithelium seems to have an important contribution in disease process. Recent histopathologic and molecular studies have shown that DNA methylation levels of SS patients compared to healthy individuals differ in epithelial cells of salivary glands and peripheral blood mononuclear cells. In the present study, we intend to analyse the epigenetic modifications of DNA in the saliva of SS patients compared to healthy controls. More specifically, salivary DNA methylation levels of selected genetic loci previously found to differ in other tissues, will be compared between SS patients and healthy controls. The study includes saliva collection from SS patients and healthy individuals, extraction of genomic DNA and methylation assessment. The epigenetic profile of each genetic locus will be correlated with SS patients’ clinical characteristics and the possibility of genetic loci with differential differences in methylation to be used as potential diagnostic biomarkers will be explored. The current study is anticipated to reveal potential biomarkers for diagnostic and therapeutic purposes, offering the advantage to utilise the easily collected and handled saliva as the main biologic material.

## BACKGROUND/INTRODUCTION

Sjögren’s syndrome is an autoimmune epithelitis of unknown aetiology. Its incidence is estimated at approximately 1:100.000 and it affects predominantly women over men, at a ratio of 15:1. The age of diagnosis is usually at the 4^th^ or 5th decade of life. Patients commonly present with mild symptoms due to inflammatory damage of the exocrine glands, such as the salivary and lachrymal glands; however, in more severe cases, internal organs such as the liver, kidneys, and lungs can be affected. Importantly, the risk of lymphoma development is significantly higher in Sjögren’s syndrome patients compared to general population. Early diagnosis could lead to better intervention and improved prognosis. Thus, disease biomarkers could provide useful tools in the clinical setting. Current diagnostic criteria include objective and subjective symptoms of dry mouth and eyes, salivary gland biopsy for assessment of lymphocytic infiltration and blood tests for the detection of antibodies against Ro/SSA and La/SSB, which are the most characteristic autoantigens of the syndrome.

Although, Sjögren’s syndrome aetiology remains largely unknown, genetic, environmental, and psychological factors are thought to contribute to its pathogenesis. Several studies support a link between epigenetic factors and the syndrome. Epigenetic modifications affect chromatin templated events in multiple ways. One of the main epigenetic modifications is methylation of DNA, which occurs mostly on the 5’ position of cytosine and in the context of CpG dinucleotides. This modification is associated with transcriptional silencing in locations such as gene promoters, repetitive sequences, and transposable elements. Several studies support altered DNA methylation in patients with Sjögren’s syndrome both in salivary epithelial cells and in B lymphocytes. Specifically, in patient samples, a global decrease of genome methylation was detected, while methylation arrays revealed a number of loci displaying hypomethylation. Decreased methylation has also been observed in the LINE1 long interspersed repeat element in Sjögren’s patient salivary glands. Although the mechanistic significance of these changes remains to be elucidated, they could provide useful molecular markers in the diagnosis of the disease. The use of saliva as a biological material provides many advantages, including the non-invasive and easy procedure of its collection. Previous studies have shown significant differences in the proteomic profile of saliva from Sjögren’s patients compared to healthy controls, supporting that this could be a useful biological material for disease diagnosis.

## AIM OF THE STUDY

We hypothesise that, similar to salivary gland tissue and peripheral lymphocytes, saliva derived from Sjögren’s patients also displays reduced methylation compared to healthy controls on many loci of interest. We aim to test this hypothesis by comparing methylation on specific loci between the two categories. The specific loci will be selected from the literature, among those that have been reported to be affected in epithelial cells or B lymphocytes. Differentially methylated loci could serve as potential biomarkers useful in the diagnosis of Sjögren’s syndrome and in monitoring of disease progression.

## RESEARCH PLAN-METHODS

To test our hypothesis, our specific aims are proposed as follows:

1) Optimisation of DNA extraction from saliva and methylation assessment. We have preliminary data supporting that good yield of good quality DNA can be obtained from saliva using standard phenol:chloroform extraction procedures. In addition, immunoprecipitation experiments using a methyl-cytosine specific antibody followed by polymerase chain reaction on known methylated and non-methylated control loci confirmed the validity of saliva-derived gDNA for such experiments.
2) Collection of saliva from Sjögren’s patients and healthy controls. This will be performed by a simple standard procedure. In parallel, we will be collecting relevant clinical information about the groups.
3) Extraction of DNA from study samples and assessment of DNA methylation on specific loci of interest. We will use bisulphite conversion followed by quantitative polymerase chain reaction. Bisulphite treatment deaminates cytosines and converts them to uracils. Methylated cytosines are not subject to conversion. Quantitative polymerase chain reaction using primers that amplify the converted or not converted product will give us the ratio of the unmethylated over methylated locus in the DNA sample.
4) Differential methylation events will also be assessed in comparison with clinical characteristics of the study individuals and results will be analysed.

## IMPACT OF STUDY

Identification of differentially methylated genomic loci of interest between Sjögren’s patients and healthy individuals will reveal potential saliva biomarkers for the diagnosis and monitoring of the disease. Saliva is a biological sample that is very easy to collect, using non-invasive procedures, making the testing of molecular markers in this material very attractive. Identification of novel biomarkers will improve disease management in many aspects.
